# Draft genome sequence of *Methylobacterium* sp. 37f isolated from forest soil

**DOI:** 10.1128/mra.00324-24

**Published:** 2024-05-20

**Authors:** Toungporn Uttarotai, Terry J. McGenity, Sawannee Sutheeworapong, Thararat Chitov

**Affiliations:** 1 Department of Highland Agriculture and Natural Resources, Faculty of Agriculture, Chiang Mai University, Chiang Mai, Thailand; 2 School of Life Sciences, University of Essex, Colchester, United Kingdom; 3 Systems Biology and Bioinformatics Laboratory, Pilot Plant Development and Training Institute, King Mongkut’s University of Technology Thonburi, Bangkok, Thailand; 4 Department of Biology, Faculty of Science, Chiang Mai University, Chaing Mai, Thailand; University of Southern California, Los Angeles, California, USA

**Keywords:** *Methylobacterium*, genome analysis

## Abstract

In this study, we report the draft genome sequence data of *Methylobacterium* sp. 37f, isolated from soil beneath *Quercus semiserrata* Roxb. in Thailand. The genome consists of 5,305,449 base pairs, with a GC content of 67.5%.

## ANNOUNCEMENT

*Methylobacterium*, a genus of Gram-negative, facultatively methylotrophic bacteria, is commonly found in soil, water, and plants (1, 2). These bacteria metabolize a broad range of hydrocarbons (3, 4), including isoprene, a climate-active gas, which *Methylobacterium* can co-metabolize (5), highlighting their ecological significance.

Strain 37f was isolated from topsoil (3 cm in depth) under a wild oak tree (*Quercus semiserrata* Roxb.) in Doi Suthep-Pui National Park, a protected forested area in Chiang Mai, Thailand. Isolation was achieved by suspending a gram of soil in 9 mL of 0.9% sterile NaCl, followed by serial dilutions and spread plating on minimal medium agar; the agar composition is detailed in reference (6). The plates were incubated at 27°C in a 10.5-L desiccator, sealed with high vacuum grease (Dow Corning, USA) to ensure an airtight environment. One milliliter of liquid isoprene was added as a potential carbon source and allowed to volatilize, forming a gaseous environment. A pink colony was picked, restreaked three times, and cultured under the same conditions until a pure culture was obtained. Genomic DNA was extracted from these agar-grown colonies using the GenElute Bacterial Genomic DNA Kit (St. Louis, MO, USA) according to the manufacturer’s guidelines.

The genome of strain 37f was sequenced by MicrobesNG at the University of Birmingham, UK. Genomic DNA libraries were prepared following the manufacturer’s protocol for the Nextera XT Library Prep Kit (Illumina, USA). The process of DNA quantification and library preparation was automated using a Hamilton Microlab STAR liquid handler. Library quantification was performed using the Kapa Biosystems Library Quantification Kit for Illumina on a Roche LightCycler 96 qPCR instrument. Sequencing of the library was carried out on the Illumina HiSeq platform, employing a 250-bp paired-end protocol. Raw reads were adapter trimmed, executed with Trimmomatic version 0.30 (7), utilizing a sliding window quality cutoff of Q15. *De novo* assembly was accomplished using SPAdes version 3.7 (8), and the quality of the assembly was assessed using QUAST version 5.0.2 (9). The completeness and contamination were assessed using CheckM version 1.2.2 (10). Lastly, the annotation of the genome was carried out using the NCBI Prokaryotic Genome Annotation Pipeline (PGAP) version 6.5 (11), with default parameters employed for all software tools utilized in this analysis. A detailed summary of the metrics for genome assembly and annotation is presented in Table 1.

**TABLE 1:** Metrics for Genome Sequencing

Genome assembly	
Genome size (bp)	5,305,449
Number of contigs	147
GC content (%) Contig N50	67.5 111,458
Contig L50	15
Genome coverage Completeness (%) Contamination (%)	30× 96.16 0.25

Genome annotation	
Number of protein-coding genes	4,812
Number of rRNA genes (5S, 16S, and 23S)	7 (3, 1, 3)
Number of tRNA genes	53

The 16S rRNA gene sequence of strain 37f exhibits 99.26% similarity to *Methylobacterium goesingense* strain iEII3 (GenBank: NR_115219), as determined by BLASTn. Calculation of the Average Nucleotide Identity (ANI) based on the BLAST algorithm (12) showed that strain 37f belongs to the genus *Methylobacterium*, sharing an ANI of 86.76% compared with *M. goesingense* strain DSM 21331 (GenBank: GCA_022179225.1).

## Data Availability

The draft genome sequence of Methylobacterium sp. 37f was deposited in GenBank under the accession number JAGHKN000000000. The BioProject, BioSample, and SRA accession numbers are PRJNA703388, SAMN18200072, and SRR13948712, respectively.
